# Pediatric Posterior Fossa ATRT: A Case Report, New Treatment Strategies and Perspectives

**DOI:** 10.3390/brainsci13050712

**Published:** 2023-04-24

**Authors:** Luca Paun, Alexandre Lavé, Gianpaolo Jannelli, Kristof Egervari, Insa Janssen, Karl Schaller, André O. von Bueren, Andrea Bartoli

**Affiliations:** 1 Division of Neurosurgery, Department of Clinical Neurosciences, Geneva University Hospitals and University of Geneva Faculty of Medicine, 1205 Geneva, Switzerland; 2 Department of Neurosurgery, Site Sainte-Anne, Groupe Hospitalier Universitaire Paris Psychiatrie et Neurosciences, Université Paris Cité, 75014 Paris, France; 3 Department of Neurosurgery, Bicêtre Hospital, Assistance Publique-Hôpitaux de Paris, Université Paris-Saclay, 94270 Le Kremlin-Bicêtre, France; 4 Department of Spine and Spinal Cord Surgery, Hôpital Pierre Wertheimer, Hospices Civils de Lyon, 69002 Lyon, France; 5 Department of Pathology and Immunology, University of Geneva, 1211 Geneva, Switzerland; 6 Division of Clinical Pathology, Geneva University Hospitals, 1205 Geneva, Switzerland; 7 Department of Pediatrics, Obstetrics and Gynecology, Division of Pediatric Hematology and Oncology, Geneva University Hospitals, 1205 Geneva, Switzerland

**Keywords:** posterior fossa, ATRT, pediatric, multimodal treatment, case report

## Abstract

Posterior fossa atypical teratoid rhabdoid tumor (ATRT) is a rare childhood tumor usually associated with a dismal prognosis. Although upfront surgical gross total resection (GTR) has classically been the first line of treatment, new multimodal treatments, including two-stage surgery, are showing promising results in terms of overall survival (OS) and complication rate. We present a case of a 9-month-old child treated with two-staged surgery and chemotherapy. When deemed risky, multimodal treatments, including staged surgeries, can be a safe alternative to reduce surgical mortality and morbidity. At 23 months old, the patient had normal global development and no major impact on quality of life. We, therefore, discuss the most recent advancements from a treatment perspective, including molecular targeting.

## 1. Introduction

Atypical teratoid rhabdoid tumors (ATRTs) are aggressive embryonal tumors with a standardized incidence of 0.15 per 100.000 inhabitants in Europe and of 75 cases per year in the US. It typically affects children younger than 3 years old, with a median age at diagnosis between 16 and 30 months [1,2,3,4].

First described in 1987 by Lefkowitz, it was initially identified as a primitive neuroectodermal tumor (PNET) [5].

Molecular analysis and technologies have allowed for a better understanding of this severe pathology, such as *SMARCB1* (INI1) gene alteration on the loss of the long arm of chromosome 22 in 95% of all cases, and *SMARCA4* (BRG1) gene alteration, located on chromosome 19p13.2, in the remaining population [3,6].

Nowadays, to diagnose this entity, INI1 or BRG1 inactivation must be found: these aberrations lead to tumorigenesis [7].

New molecular discoveries have led to the current classification proposed in 2020 that divides ATRT into three main molecular subgroups, notably ATRT-TYR, ATRT-SHH, and ATRT-MYC [8,9,10,11,12].

- ATRT-TYR typically arises in the posterior fossa, in which *SMARCB1* is inactivated most often by heterozygous mutations completed by whole or partial chromosome 22 loss removing the second allele. It shows overexpression of tyrosinase [13];
- ATRT-SHH (Sonic Hedgehog) occurs as either supra- (type 1) or infratentorial (type 2), with frequent compound heterozygous *SMARCB1* point mutations and overexpression of proteins in the SHH and Notch signaling pathways;
- ATRT-MYC is more commonly supratentorial with *SMARCB1* deletions and *MYC* and *HOX* genes overexpression.

The introduction of this classification aims to unify the current knowledge and future research on targeted therapies for ATRT [12,14].

ATRT treatment still relies on surgery (ideally gross total resection (GTR) with preserved neurological function) in order to time adjuvant treatments, especially radiotherapy that can lead to harmful effects on brain development [1,15,16,17].

Even with trimodal treatment (consisting of surgery, induction chemotherapy, and consolidation treatment) depending on central nervous system (CNS) dissemination, metastatic (M+) ATRT OS at 3 years remains at 25% [18].

Postoperative morbidity can be particularly high in posterior fossa ATRT, including hydrocephalus (HCP) in up to 80% of all cases, pseudomeningocele in up to 25%, as well as posterior fossa syndrome, meningitis, and lower cranial nerve palsies [19,20,21].

Tailored multistaged and multimodal treatments appear to be a safe strategy to control the disease and preserve the functional outcome.

In this paper, we present a case of a 9-month-old female child with a posterior fossa ATRT treated with a partial debulking, neoadjuvant chemotherapy, and subsequent resective surgery. Secondly, we present a narrative review of the most recent relevant literature. 

## 2. Materials and Methods

Demographic, clinical, radiological, and surgical information on the patient was retrospectively collected at Geneva University Hospitals. Written informed consent was obtained from the parents.

The detailed information about the pathologic characteristics was obtained from Geneva University Hospitals. We conducted restricted research using the keywords “Posterior Fossa” AND/OR “ATRT”, “EU-RHAB” AND/OR “Posterior Fossa”, “MULTISTAGE SURGERY” AND/OR “ATRT” in November 2022 in the following databases: PubMed/Medline and Google Scholar. The CARE guidelines were followed. 

The first author independently screened all titles and abstracts, and full-text copies of all relevant articles were obtained without the exclusion of pertinent studies. Research was collected from 1980 on in order to avoid obsolete pathological and molecular data. In addition, CNS ATRT was recognized as a separate entity and added to the World Health Organization (WHO) tumor classification of tumors in 2000 as a grade IV embryonal tumor [22]. We excluded all non-English studies. In the case of discrepancy, the senior author (AB) arbitrated until a consensus among the authors was reached. No statistical analysis was performed.

## 3. Case Report

**Figure 1:**
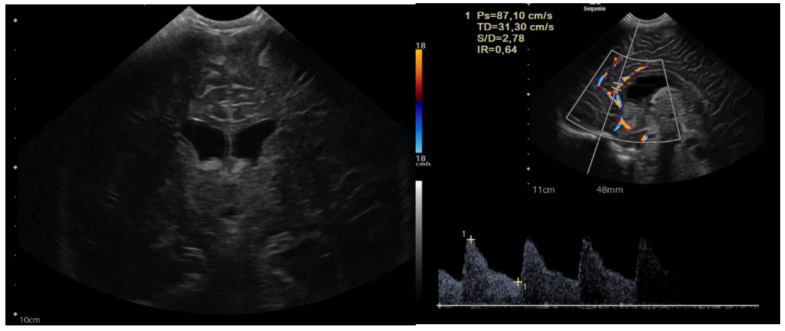
Sagittal and coronal US showing dilated lateral ventricles. Echocolordoppler showed augmented resistance and pulsatility indexes, speaking in favor of augmented intracranial pression.

The child was 9 months old with eutocic delivery at 40 gestation weeks, and, according to the parents started to develop ataxia and hypotonia at 6 months old. She developed repeated morning and orthostatic vomiting. The head circumference was at the 98th percentile (46.5 cm), and a cerebral ultrasound (US) showed enlarged lateral ventricles (Figure 1).

**Figure 2:**
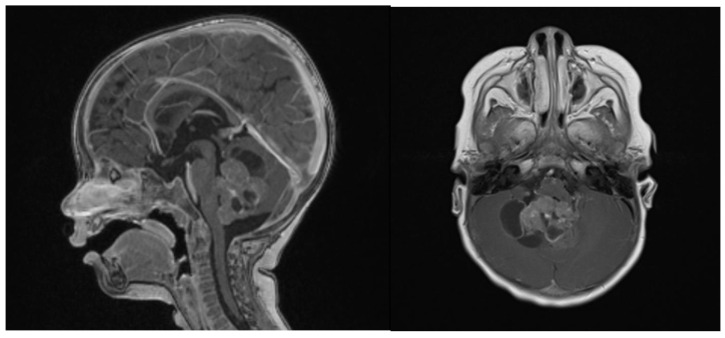
Sagittal and axial gadolinium T1 MR sequence showing the exophytic mass occluding CSF diversion at the IV ventricle, compressing the brainstem and adjacent right cerebellar hemisphere.

Clinical examination did not show any anterior fontanel bulging nor trismus. Whole brain and spine magnetic resonance imaging (MRI) was performed, showing a heterogeneous cystic-hemorrhagic lesion, partially calcified, in the posterior fossa, Measuring 46 × 34 × 49 mm and obstructing the IV ventricle and right Luschka foramen (Figure 2).

**Figure 3:**
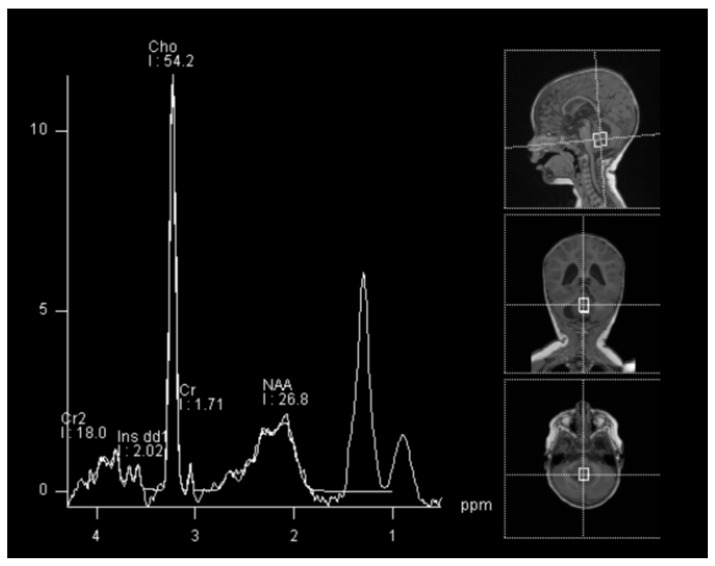
Spectroscopy analysis showing an augmented rate of choline, in favor of a malignant lesion.

In spectroscopy sequences an elevated choline peak and low N-Acetylaspartate (NAA) and creatine, were found, suggesting an ATRT (Figure 3). No leptomeningeal dissemination was found. 

Multidisciplinary consensus opted for upfront GTR. At the time of the first surgery, the child weighed 8 kg and measured 76 cm. Surgery, performed by an experienced senior microneurosurgeon, had to be aborted due to extreme blood loss from an intrinsically highly vascular tumor, and an external ventricular drainage (EVD) was left in place. A 460 mL blood transfusion, composed of red blood cells (RBC) and platelets (PLT), was given. 

After uneventful postoperative surveillance in the pediatric intensive care unit (PICU), the EVD was converted into a ventriculoperitoneal shunt (VPS) since the obstructive HCP had not been resolved. Moreover, foreseeing the need for an intrathecal treatment, and to avoid any manipulation of the VPS, an additional intraventricular Ommaya reservoir was left in place.

**Figure 4:**
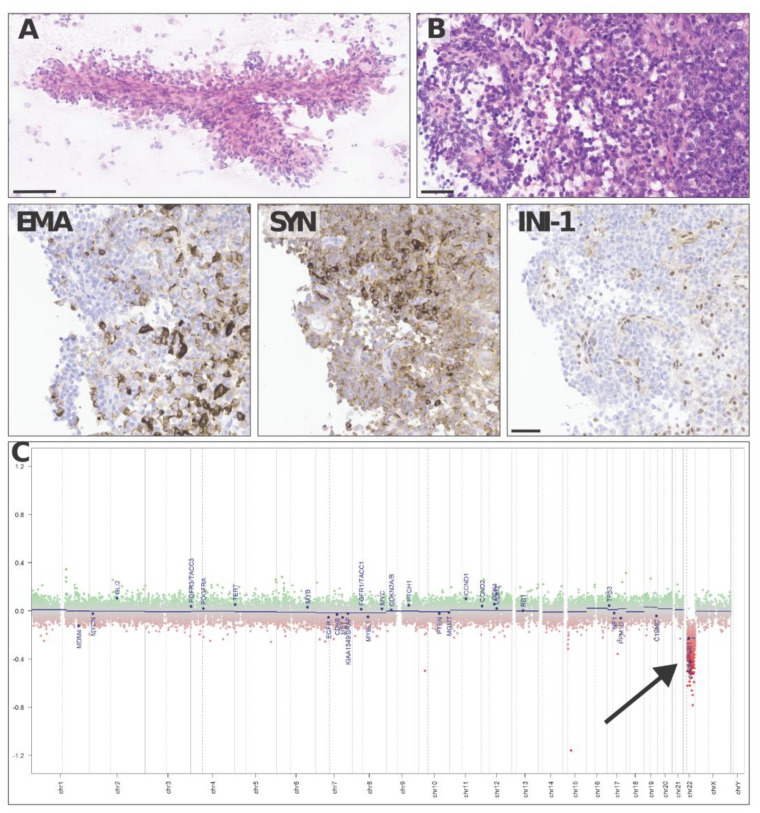
Representative haematoxylin–eosin images of an intraoperative cytologic smear (**A**) andparaffin-embedded histological sections (**B**) show a densely cellular solid–papillary tumor, made up of tumor cells positive with epithelial membrane antigen (EMA) and synaptophysin (SYN) and showing loss of INI-1 (SMARCB1) on immunostains. Copy number variation predictions (**C**) show a flat profile with a heterozygous loss of 22q harboring SMARCB1 (arrow in **C**). Scale bars = 100 µm (**A**) and 50 µm (**B**, EMA, SYN, INI-1).

Neuropathological analysis found a highly vascularized, densely cellular tumor showing a predominantly papillary architecture with solid areas (Figure 4A,B). Tumor cells often displayed an abundant eosinophilic cytoplasm and large vesicular nuclei with nucleoli. The tumor cells were in part positive for epithelial membrane antigen (Figure 4 EMA) and synaptophysin (Figure 4 SYN) and showed a loss of INI-1 (SMARCB1, Figure 4 INI-1) expression on immunostains, consistent with the diagnosis of an atypical teratoid rhabdoid tumor (ATRT), CNS WHO grade 4. Methylation-based classification using the Brain Tumor Classifier (v11b4, DKFZ, Heidelberg) confirmed the diagnosis and gave a match with the methylation class ATRT, subclass TYR. As expected, copy number variation (CNV) predictions showed a flat genomic profile with a heterozygous loss of 22q harboring SMARCB1 (Figure 4C).

The multidisciplinary pediatric CNS tumor board opted for chemotherapy following SIOPE ATRT01 (EudraCT Number: 2018-003335-29)/EU-RHAB Registry protocol [23]. As induction chemotherapy, the patient received six courses of chemotherapy, as used in the EU-RHAB protocol (DOX (doxorubicin), ICE (ifosfamide, carboplatin, and etoposide), and VCA (vincristine, cyclophosphamide, and actinomycin D), with intraventricular methotrexate (MTX)). 

**Figure 5:**
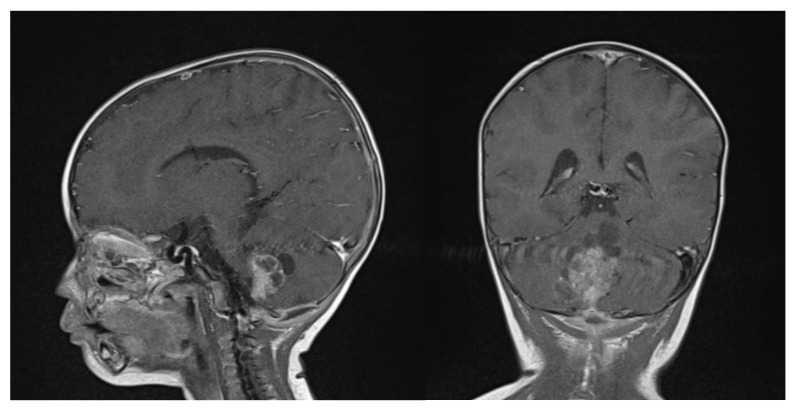
Gadolinium T1 MR sagittal and axial sequences at 6 weeks post-chemotherapy induction. ATRT volume diminished from 43.7 cm^3^ to 16.6 cm^3^.

At 6 weeks after starting induction chemotherapy (after 3 courses of induction chemotherapy), craniospinal MRI showed a partial response to the treatment, with a >50% volume shrinking and no leptomeningeal dissemination, as assessed by an experienced senior neuroradiologist consultant (Figure 5). Considering the tumor volume reduction and the good general condition, a second-look surgery was offered in an attempt to achieve a GTR.

**Figure 6:**
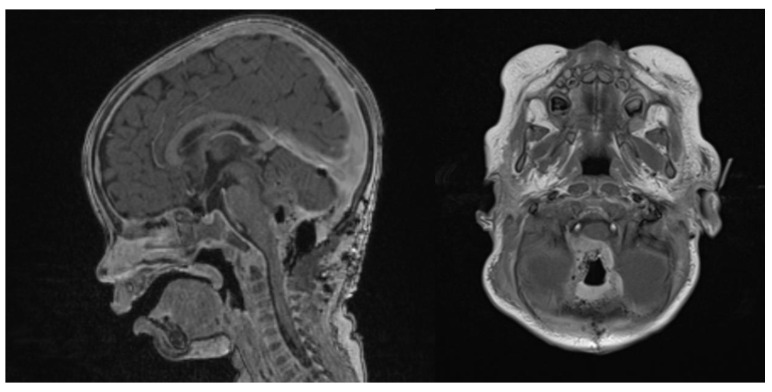
Second surgery: postoperative Gadolinium T1 and substraction sequences showing a GTR without complications.

The second-look surgery was uneventful. The child weighed 9 kg and was 76 cm tall. A millimetric residual tissue was left in place, infiltrating the floor of the IVth ventricle at the bulbo-pontine junction on the right. Blood loss was estimated to be around 50 mL, whilst the patient was transfused with 2 RBC and of 1 PLT concentrate. A new postoperative MRI confirmed a millimetric enhancement at the right bulbo-pontine junction (Figure 6).

Then 3 additional courses of chemotherapy were applied (with stem cell apheresis). Thereafter, 3 courses of high-dose chemotherapy, at least 28 days apart, each consisting of carboplatin, thiotepa, and autologous stem cell reinfusion on day 0 were performed.

During the follow-up, the patient status was assessed by different consultants, including a pediatric neurosurgeon, pediatric oncologists and pediatric neuroradiologists. All treatment decisions were based on a consensus from the pediatric tumor board. Until now, overall follow-up lasted 14 months. 

**Figure 7:**
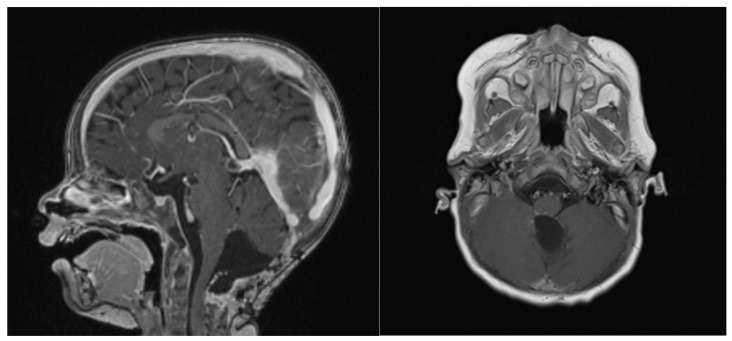
14 months post-operative gadolinium T1 sagittal and axial sequences, showing no signs of recurrence.

At the last follow-up at 23 months old, the patient did not show any sign of recurrence, with stable enhancing tissue on the floor of the IVth ventricle. Clinical examination was normal, as was global development (as stated by a consultant infantile neuropsychiatrist) (Figure 7). No adverse or unanticipated events were assessed. In this particular situation, local proton therapy (according to the ACNS0333 trial) was recommended [24].

## 4. Discussion

ATRT is an aggressive tumor with poor prognosis and its outcome depends on age, location, and biological and histological characteristics [16]. 

Considering its rare occurrence, posterior fossa ATRT remains difficult to cure, and even with multimodal treatments, the prognosis remains dismal, with a mean survival between 6 and 11 months [1,15].

In the recent literature, the mean OS for ATRT patients does not exceed 12 months, and available series consider mixed cases of supra- and infra-tentorial cases (Table 1). Similarly to most brain tumors in the first two years of life, the extent of surgical resection is positively correlated with overall outcome. GTR cannot be always achieved due to the delicate surrounding anatomical structures, the voluminous size and the hemorrhagic nature of many infantile tumors [13]. Herein, we report a case with the highest OS, characterized by a good quality of life and no major impact on global development. 

Historically, the mortality rate in infants undergoing surgery for primary brain tumors in the first two years of life has undoubtedly exceeded that for older children, ranging from 7.3% to 33% depending on the nature of the tumor, tumor size, age at surgery, surgical blood loss, and actual definition of surgery-related death [35,36,37,38,39]. However, advances in preoperative planning and imaging, microneurosurgical techniques, pediatric neuroanesthesia, and dedicated postoperative PICU can achieve very low mortality rates [16,40].

When upfront GTR is deemed to be high risk, partial removal and a multistage approach can be offered as safe alternatives. 

Multi-stage approaches were first described for anatomically challenging complex lesions, mostly involving tumor removal and/or vascular exclusion for arteriovenous malformation (AVM) [41].

Dandy, in 1925, described a vestibular schwannoma operated on in a two-stage surgery; Spetzler and his team described, in 1986, an AVM surgical resection anticipated by embolization [41,42,43]. 

Multi-staged surgery approaches have evolved into multistage treatment involving non-surgical treatments such as radiosurgery, chemotherapy, and targeted therapy.

Until now, the only case reported in literature concerning a two-staged surgical treatment of a supra- and infratentorial ATRT tumor was described by Foreman et al. in 2016, in order to minimize surgical morbidity and the risk of postoperative complication [44].

Considering ATRT’s anatomical and pathological complexity, clinical decisions should take into account the family’s expectations, molecular profile, long-term sequelae, and expert network pathology centralization. 

Together with the safest achievable GTR, methylation profiling data and tailored treatment are considered key factors to achieve longer OS, longer progression-free survival (PFS), and acceptable overall quality of life [45].

Initial attempts to unify and categorize ATRT by immunohistochemistry were made by Torchia et al., who created two subgroups based on the existence (Group 1) or absence of ASCL1 overexpression (Group 2) [10]. This classification was rapidly adapted and expanded by the same authors with methylation array profiling, allowing to divide ATRT as described in the introduction [11]. Finally, Han et al., using the same methodology, created 3 subgroups based on the overexpression of different genes, such as ASCL1, BMP4, and ACTL6A [9,12]. Currently, the latter is the most often used to categorize patients in order to tailor specific molecular treatments to them.

Different MRI studies are demonstrating correlations between specific radiological findings and the three different molecular subgroups [46].

Following this taxonomy, a St. Jude institute study group compared two multi-institutional clinical trials with the aim of reporting molecular groups’ relevance to clinicopathologic features and the survival of children affected by ATRT.

In their series, they profiled 64 ATRT cases as 21 TYR (33%), 30 SHH (47%), and 13 MYC (20%): the median age at diagnosis was lower in the SHH subtype and higher in the MYC subtype. They demonstrated that among infants, ATRT-TYR had the best OS, whilst ATRT-SHH was associated with metastasis and consequent inferior outcomes [3]. These findings have led to the adaptation of neoadjuvant protocol treatment.

In the case of SMARCB1 mutations, there is no direct target, whilst other upregulated pathways can be sensitive to different molecules [7]. Small molecular inhibitors such as alisertib (Aurora Kinase A inhibitor, NCT02114229), ribociclib (CDK 4/6 inhibitor, NCT03434262), and tazemetostat (EZH2 inhibitor, NCT02601937) have been introduced in trials, and, in some ATRT series demonstrated good results in terms of volume regression [47,48].

Moreover, ATRT-SHH was found to be sensitive to histone methyltransferase G9a (UNC0638), whilst ATRT-TYR and ATRT-MYC were found to be sensitive to PDGFRB enhancers (nilotinib and dasatinib) and to bone morphogenetic protein (BMP) [7,49,50].

Some of the new molecular inhibitors can be difficult to offer in the pediatric population: new types of orthotopic xenograft models have been created to study in vitro tumors’ responses to new potential treatments [51]. Other preclinical investigations are being conducted concerning marizomib, a small-molecule proteasome-inhibitor that permanently inactivates all 3 enzymatic functions (trypsin-like, chymotrypsin-like, and caspase-like) of the proteasome and the immunoproteasome. Moreover, this molecule can cross the blood–brain barrier (BBB), inducing intracellular oxygen species, and can overcome existing resistance to other molecules (such as bortezomib (BTZ) and carfilzomib (CFZ), used in the past in glioblastomas, medulloblastomas, and rhabdomyosarcomas) [52].

Improvements in molecular targeted therapy will include molecules able to simultaneously distinguish tumor cells from normal cells, as well as molecules able to avoid escaping mechanisms and to cross the BBB [53,54].

Other new cell engineering techniques are being deployed in clinical trials, such as chimeric antigen-receptor T-cells (CAR-T), which targets B7-H3 in ATRT-MYC in mice models and is showing promising results [55]. 

These new engineering techniques seem to open new pathways to effective treatments implementing OS, but specific attention should be paid to short- and long-term direct drug toxicity, as well as neurological and reproductive sequelae [56,57].

Tumoral heterogeneity still remains a pivotal point in effective treatment [58]. Finally, a wider understanding of the molecular composition of ATRT subgroup types will help to inform tumor-specific treatment in the future [11].

Considering the rarity of this pathology, shared expertise has allowed research to widen and improve ATRT knowledge: the EU-RHAB Registry identified clinical factors influencing positive prognosis (such as age older than 3 years, radiotherapy use and achievement of complete remission) or negatively (such as male sex, presence of synchronous tumors, and germline mutations) [23,59]. Multicentric efforts and databases will be required in order to achieve greater clinical impact on those patients. 

Lastly, this global expertise should be shared as much as possible with children’s families: while in the past clinical decisions were often made solely by the surgeon/oncologist and accepted by the family, nowadays, the decision process is shifting towards an open discussion about risks and actual expectations before a common decision is made with the patient and his/her family [60].

## 5. Conclusions

Pediatric posterior fossa ATRT is an heterogenous disease with unfavorable survival. GTR contributes in a relevant manner to the local control. When deemed risky, multimodal treatments, including staged surgeries, can be a safe alternative to reduce surgical mortality and morbidity, and to guarantee a good quality of life. New surgical technologies and targeted molecular therapies show promising results and could be integrated in such a multimodal approach after proving their efficacy. Further efforts should be put into multicentric studies.

## Figures and Tables

**Table 1:** Review of the literature on the most recent ATRT mixed series (supra- and/or infratentorial), underlining mean OS.

Author	Title	Year	Journal	OS (Months)
Silva et al. [25]	Atypical teratoid rhabdoid tumors (ATRTs)—a 21-year institutional experience	2023	*Child’s Nervous System*	11
Picariello et al. [16]	Posterior Fossa Tumors in the First Year of Life: A Two-Centre Retrospective Study	2022	*Diagnostics*	NA
Guo et al. [26]	Atypical Teratoid/Rhabdoid Tumor of the Central Nervous System in Children: Case Reports and Literature Review	2022	*Frontiers in Surgery*	12
Richards et al. [21]	Outcomes with respect to extent of surgical resection for pediatric atypical teratoid rhabdoid tumors	2020	*Child’s Nervous System*	NA
Din et al. [27]	Atypical Teratoid/Rhabdoid Tumor of Brain: a Clinicopathologic Study of Eleven Patients and Review of Literature	2017	*Asian Pacific Journal of Cancer Prevention*	NA
Tomita et al. [28]	Tumors of the superior medullary velum in infancy and childhood: report of 6 cases	2013	*Journal of Neurosurgery: Pediatrics*	7.5
Hasan et al. [29]	Treatment-related morbidity in atypical teratoid/rhabdoid tumor: multifocal necrotizing leukoencephalopathy	2011	*Pediatric Neurosurgery*	7
Mohapatra et al. [30]	Histological and immunohistochemical characterization of AT/RT: a report of 15 cases from India	2010	*Neuropathology*	NA
Rahmat et al. [31]	A child with atypical teratoid/rhabdoid tumor of the posterior cranial fossa	2008	*Singapore Medical Journal*	4
Parmar et al. [32]	Imaging findings in primary intracranial atypical teratoid/rhabdoid tumors	2006	*Pediatric Radiology*	12
Chen et al. [33]	Atypical teratoid/rhabdoid tumors of the central nervous system: management and outcomes	2005	*Neurosurgical Focus*	18.5
Inenaga et al. [34]	A fourth ventricle atypical teratoid/rhabdoid tumor in an infant	2003	*Brain Tumor Pathology*	NA

## Data Availability

All data are available in the text.

## Abbreviations

ATRT	Atypical teratoid rhabdoid tumor
GTR	Gross total resection
OS	Overall survival
PNET	Primitive neuroectodermal tumor
CNS	Central nervous system
HCP	Hydrocephalus
WHO	World Health Organisation
US	Ultrasound
MRI	Magnetic resonance imaging
NAA	N-Acetylaspartate
EVD	External ventricular drainage
RBC	Red blood cell
PLT	Platelets
PICU	Pediatric intensive care unit
VPS	Ventriculoperitoneal shunt
CNV	Copy Number Variation
MTX	Methotrexate
AVM	Arteriovenous malformation
PFS	Progression-free survival
BBB	Blood-Brain barrier
CAR	Chimeric antigen receptor
